# Blue Light Mediates Chloroplast Avoidance and Enhances Photoprotection of Vanilla Orchid

**DOI:** 10.3390/ijms21218022

**Published:** 2020-10-28

**Authors:** Swee-Suak Ko, Chung-Min Jhong, Yi-Jyun Lin, Ching-Yu Wei, Ju-Yin Lee, Ming-Che Shih

**Affiliations:** 1 Academia Sinica Biotechnology Center in Southern Taiwan, Tainan 741, Taiwan; amo2175111@gmail.com (C.-M.J.); hbabyi@gate.sinica.edu.tw (Y.-J.L.); 2 Agricultural Biotechnology Research Center, Academia Sinica, Taipei 115, Taiwan; 3 National Chiayi University Department of Forestry and Natural Resources, Chiayi 600, Taiwan; minny881221@mail.com; 4 National Taiwan University Department of Horticulture and Landscape Architecture, Taipei 10617, Taiwan; turtlejoanna@gmail.com

**Keywords:** *Vanilla planifolia*, high irradiance, photoprotection, blue light acclimation, chlorophyll fluorescence

## Abstract

Vanilla orchid, which is well-known for its flavor and fragrance, is cultivated in tropical and subtropical regions. This shade-loving plant is very sensitive to high irradiance. In this study, we show that vanilla chloroplasts started to have avoidance movement when blue light (BL) was higher than 20 μmol m^−2^s^−1^ and significant avoidance movement was observed under BL irradiation at 100 μmol m^−2^s^−1^ (BL100). The light response curve indicated that when vanilla was exposed to 1000 μmol m^−2^s^−1^, the electron transport rate (ETR) and photochemical quenching of fluorescence (qP) were significantly reduced to a negligible amount. We found that if a vanilla orchid was irradiated with BL100 for 12 days, it acquired BL-acclimation. Chloroplasts moved to the side of cells in order to reduce light-harvesting antenna size, and chloroplast photodamage was eliminated. Therefore, BL-acclimation enhanced vanilla orchid growth and tolerance to moderate (500 μmol m^−2^s^−1^) and high light (1000 μmol m^−2^s^−1^) stress conditions. It was found that under high irradiation, BL-acclimatized vanilla maintained higher ETR and qP capacity than the control without BL-acclimation. BL-acclimation induced antioxidant enzyme activities, reduced ROS accumulation, and accumulated more carbohydrates. Moreover, BL-acclimatized orchids upregulated photosystem-II-associated marker genes (*D1* and *PetC*), *Rubisco* and *PEPC* transcripts and sustained expression levels thereof, and also maximized the photosynthesis rate. Consequently, BL-acclimatized orchids had higher biomass. In short, this study found that acclimating vanilla orchid with BL before transplantation to the field might eliminate photoinhibition and enhance vanilla growth and production.

## 1. Introduction

Vanilla orchids make up about 110 species in the orchid family (*Orchidaceae*) and boast the family’s only edible fruit. Vanilla orchids are distributed between 27 °N and 27 °S on all continents [1]. *Vanilla planifolia*, the most popular cultivated species of vanilla, known as “Bourbon” or “Mexican” vanilla, is native to the tropical forests of Central America and Mexico and was historically cultivated by the peoples of Mesoamerica. Vanilla pods produce vanillin, which is a fragrant compound with high economic value. Today, market demand for natural vanillin is increasing, and vanilla orchid is cultivated in many tropical and subtropical zones [2]. Vanilla orchid performs crassulacean acid metabolism (CAM) photosynthesis. Photosynthetically active radiation (PAR) is the portion of the light spectrum that is able to be utilized for photosynthesis. For a CAM plant, PAR intensity during the day affects the rate of mobilization of organic acids from the vacuole (Phase III) [3]. In addition, light intensity during the day also affects CO_2_ absorption during the night (Phase I), thus affecting the abundance of carbohydrate production through the Calvin cycle. Vanilla orchid is a shade-loving plant that is very sensitive to high radiation. For vanilla orchid production, support trees should have a dense canopy to provide 70% to 80% shade [4]. A high light of 67% relative illumination inhibits vanilla growth, reduces Fv/Fm, and decreases CO_2_ uptake. It has also been found that vanilla has higher photosynthesis and biomass growth under intermediate levels of radiation of 17% to 31% relative illumination [5].

Plants are known as photoautotrophs because they have the ability to conduct photosynthesis, which synthesizes glucose from carbon dioxide and water using light energy. Photosynthesis is a comprehensive pathway that consists of the light-dependent (photosystem (PS)) and light-independent (Calvin cycle) pathways. Light reactions take place in the chloroplast thylakoid membrane for the splitting of two water molecules into one oxygen molecule and four protons and electrons. Then, the protons and electrons are transferred through the thylakoid membrane to create the energy-carrying molecules adenosine triphosphate (ATP) and nicotinamide adenine dinucleotide phosphate (NADPH). ATP and NADPH are then used by RuBP carboxylase/oxygenase for the Calvin cycle to convert CO_2_ into carbohydrates [6]. PSII and PSI are located in the thylakoid membranes, and they are very sensitive to high light-induced oxidative stress. If PAR is in excess, the electron transport of PSII is inhibited, and its core protein structure is damaged. Sequentially, PSI can be impaired if the electron transport of PSII is restricted [7]. Excess light causes ROS toxicity and oxidative stress to the thylakoid membrane and other essential components of chloroplasts, finally blocking photosynthesis and causing photo-oxidative damage [8].

To prevent photoinhibition and to acquire acclimation to light-fluctuating environments, plants have developed direct and indirect mechanisms for sensing and reacting to the high light irradiation [9]. Photoprotection mechanisms include regulation of light absorption and dissipation of excess light energy. Plants can change their leaf area and leaf angle, initiate chloroplast avoidance, and decrease the size of their light-harvesting complex (LHC) antenna. Blue light photoreceptors, called phototropins (phot1 and phot2), play an essential function in chloroplast movement [10]. A recent study showed that blue light induces chloroplast movement in several monocot C_3_ plants [11]. In CAM moth orchids, when blue light irradiation is above 25 μmol m^−2^s^−1^, chloroplasts start to show avoidance movement and significant chloroplast avoidance has been found in the strong blue light of 100 μmol m^−2^s^−1^ [12]. When extra light has been absorbed, the plant will dissipate the excessive light energy through thermal dissipation via nonphotochemical quenching (NPQ) [7,13]. Plants also have other photoprotective modes such as nonphotochemical quenching of fluorescence (qN) and photochemical quenching of fluorescence (qP), which can be early signs of photoinhibition [14]. D1 (PsbA), a photosystem II reaction center protein, is a target of photodamage under excess light. Degradation and turnover rate of the new synthesis of D1 protein affect the photoprotection level [15]. Overexpressing D1 protein in *Arabidopsis*, tobacco and rice increased photosynthesis efficiency and significantly enhanced crop yield [16]. In addition, overexpressing Rieske Fes protein (PetC), a subunit of the Cytochrome b6f complex, increased D1 protein and enhanced photosynthesis efficiency of PSI and PSII, consequently improving *Arabidopsis* growth and seed yield [17]. Moreover, overexpression of the Rieske FeS protein increases C4 photosynthesis in *Setaria viridis* [18]. Clearly, increasing and manipulating the photosynthesis rate and combining several promising photosynthetic traits might enhance plant growth and crop yield [19,20].

Reactive oxygen species (ROS), such as singlet oxygen and H_2_O_2_, are generated as byproducts of photosynthesis and play important roles as signals involved in responses to excess light [9]. Plants turn on ROS scavenging enzymes such as superoxide dismutase (SOD), catalase (CAT), and ascorbate peroxidase (APX) to detoxify ROS molecules [21]. Moreover, they have antioxidative defense mechanisms to operate reduction–oxidation (redox) homeostasis and maintain ROS efficiently [8].

To date, basic studies on vanilla orchid are scarce. In our previous study, we showed that blue light mediated the photoacclimation of moth orchid [22]. We, therefore, hypothesized that blue light may also induce the photoacclimation of vanilla orchid. The objectives of this study are (1) to learn whether blue light can induce chloroplast avoidance in vanilla, (2) to detect the Chl F parameters of vanilla under various light and temperature regimes, (3) to test whether blue light pretreatment can induce photoacclimation and enhance photoprotection under high irradiation, and (4) to investigate ROS, sugar content, and gene expression patterns after BL-acclimation.

## 2. Results

### 2.1. Blue Light Induces Chloroplast Movement in Vanilla Orchid

**Figure 1:**
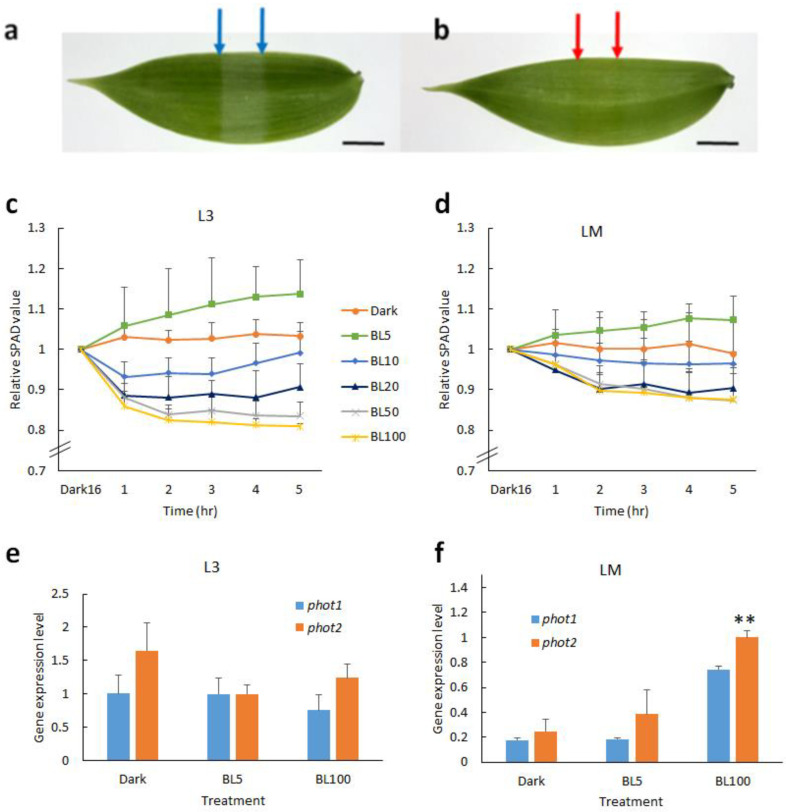
Effect of light on vanilla orchid chloroplast movement and gene expression patterns of phototropins. Slit assay showed that vanilla orchid chloroplast movement is dependent on blue light. Young leaves (L3) were dark-adapted for 4 h and then exposed overnight to monochromic LED blue light at 50 μmol m^−2^s^−1^ (**a**) and red light at 50 μmol m^−2^s^−1^ (**b**), respectively. Blue and red arrows indicate the leaf area exposure to blue or red light for slit assay chloroplast movement. Scale bar, 1 cm (**a,b**). (**c**) SPAD assay chloroplast movement of young vanilla leaves (L3) in response to various blue light intensities. (**d**) Chloroplast movement of mature vanilla leaves (LM). Quantitative RT-PCR indicated the gene expression patterns of *phot1* and *phot2* in the dark and under BL5 and BL100, respectively, in young leaves (L3) (**e**) and mature leaves (LM) (**f**). Error bars represent the standard error of the mean (*n* = 3) (**c**–**f**).

Vanilla orchid is sensitive to high irradiation that causes photoinhibition and leads to sunburn phenotype in the field (Figure S1). It is believed that chloroplast avoidance can reduce photodamage. First, to understand whether vanilla orchid chloroplast movement is dependent on blue light (BL) or red light, we exposed vanilla orchid leaves (L3) to monochromic LED red light (RL, λmax = 660 nm) and blue light (BL, λmax = 450 nm), respectively, at a light intensity of 50 μmol m^−2^s^−1^ for 5 h. Slit assay results indicated that BL-treated leaves have a light green color, but RL-treated leaves do not (Figure 1a,b). Hence, chloroplast movement is dependent on blue light in vanilla orchid. Next, we compared the chloroplast movement of young (L3) and mature (LM) leaves at various BL intensities. Vanilla orchid leaves were dark-adapted overnight then exposed to BL of 0, 5, 10, 20, 50, and 100 μmol m^−2^s^−1^, respectively, for 5 h, and Soil Plant Analysis Development (SPAD) data were recorded every hour. ANOVA analysis data indicated that BL intensity significantly affected the chloroplast movement of L3 and LM, but the interaction of BL × Time was of no significant difference (Table 1). Under continuous dark conditions, the relative SPAD value was stable and close to one (baseline), indicating that chloroplasts did not move. When L3 leaves were irradiated with BL5, the relative SPAD value was greater than one, implying that the orchid had chloroplast accumulation movement (Figure 1c). Under BL10, chloroplasts tended to show avoidance in the first 3 h, then returned back to baseline. When irradiated ≥BL20, chloroplasts started to show avoidance movement, and significant chloroplast avoidance was observed when leaves were exposed to BL100 (Figure 1c). Compared to young leaves, mature leaves (LM) had a narrower range SPAD curve, indicating that mature leaves had less chloroplast movement (Figure 1d). These data indicated that vanilla orchid young leaves are more sensitive to BL irradiation than the mature leaves. Both young and mature leaves of vanilla orchid have chloroplast avoidance movement when exposed to ≥BL20 irradiation. We collected leaf samples after 4 h exposure to BL5, BL100, and dark control, isolated RNAs, and detected gene expression of *phot1* and *phot2*. qRT-PCR results indicated that young leaves (L3) expressed a similar amount of *phot1* and *phot2* at BL5, and *phot2* was expressed at a higher level than *phot1* in the dark and under BL100 (Figure 1e). For mature leaves (LM), dark conditions resulted in the lowest gene expression levels of *phot1* and *phot2*; the second-highest levels were at BL5, whilst the highest gene expression levels of *phot1* and *phot2* were found in BL100. It was found that *phot2* was expressed at higher levels than *phot1* in the dark and under BL5 and BL100 in the mature leaves of vanilla orchid (Figure 1f).

### 2.2. Effect of Light and Temperature on Chl F of Vanilla Orchid

**Figure 2:**
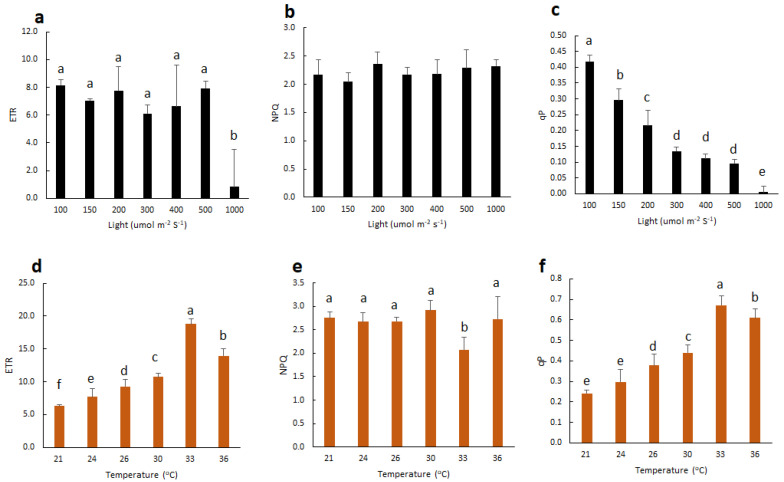
Effect of light intensities and temperatures on the Chl F parameters of ETR, NPQ, and qP. (**a**–**c**) Vanilla leaves were exposed to light regimes of 100 to 1000 μmol m^−2^s^−1^. Fluorescence leaf chamber temperature was set at 26 °C. (**d**–**f**) Vanilla leaves were exposed to various leaf chamber temperatures, from 21 to 36 °C. Light was irradiated with 150 μmol m^−2^s^−1^. Statistical significance was determined by the ANOVA procedure, followed by Duncan’s multiple range test. The same letter indicates no significant difference between treatments (*p* < 0.05). Error bars represent the standard error of the mean (*n* = 3).

To understand how light intensity affects vanilla orchid photosynthesis, we measured the Chl F parameters of vanilla orchid leaves exposed to light (composed of 90% red light and 10% blue light), with various light intensities at 100, 150, 200, 300, 400, 500, and 1000 μmol m^−2^s^−1^, respectively. Results indicated that high light at 1000 μmol m^−2^s^−1^ significantly reduced ETR (Figure 2a). NPQ showed no significant difference between various light intensities (Figure 2b). The qP peaked at 100 μmol m^−2^s^−1^, then gradually decreased when light intensities were higher than 150 μmol m^−2^s^−1^ and decreased to near zero at 1000 μmol m^−2^s^−1^ (Figure 2c).

We then fixed leaf chamber light intensity at 150 μmol m^−2^s^−1^ and set the temperature to 21, 24, 26, 30, 33, or 36 °C and detected Chl F parameters. Results indicated that 21 °C had the lowest ETR and qP but increased when the temperature increased. Vanilla orchid tended to have the highest ETR and qP at 33 °C, then gradually decreased again when the temperature increased up to 36 °C (Figure 2d,f). Vanilla orchid had the lowest NPQ at 33 °C (Figure 2e).

### 2.3. Extended BL-Acclimation Duration Enhances Chl F Capacity

The reduction of photoinhibition of moth orchid induced by blue light acclimation was recently reported [22]. Similar to moth orchid, vanilla orchid is a shade-loving CAM plant that is sensitive to high light (Figure 2). We hypothesized that BL-acclimation might enhance the photoprotection of vanilla orchid. To determine the suitable BL treatment duration for inducing photoacclimation, we treated vanilla orchid with 100 μmole m^−2^s^−1^ blue light (λmax = 450 nm) for 0, 4, 8, or 12 days. In order to investigate the effect of light intensity on the Chl F capacity of vanilla orchid, we set the fluorescence leaf chamber with light (composed of 90% red light and 10% blue light) intensities of 150, 500, and 1000 μmol m^−2^s^−1^, respectively, and detected the Chl F parameters. Analysis of variance (ANOVA) statistical analysis indicated that all the Chl F parameters had a significant difference between the various BL-acclimation time courses. It is found that light intensities were significantly different for ETR, NPQ, qP, and qN parameters. There was also a significant difference in BLday × light interaction for the parameters of ETR and qP (Table 2). For the control without BL-acclimation, orchids at the normal light intensity of 150 μmol m^−2^s^−1^ had higher ETR and qP values but a lower amount of NPQ and qN than leaves exposed to high light of 1000 μmol m^−2^s^−1^. When vanilla orchid was BL-acclimated for 4 days and irradiated with 150 μmol m^−2^s^−1^, vanilla leaves had significantly higher ETR and qP than the control without BL-acclimation, but, with this time course, ETR and qP were still low when exposed to 1000 μmol m^−2^s^−1^ (Table 2), implying that BL-treatment for four days does not provide complete acclimation in vanilla orchid. Surprisingly, after BL treatment for 8 days or 12 days, vanilla leaves tended to increase Chl F capacity even when exposed to high light of 1000 μmol m^−2^s^−1^ (Table 2). Clearly, vanilla orchid acquired photoacclimation due to prolonged BL treatment. These data also indicated that the high intensity of 1000 μmol m^−2^s^−1^ had lower Chl F parameters than the low light intensity of 150 μmol m^−2^s^−1^. Moreover, extended BL-acclimation for 8 or 12 days enhanced Chl F capacity under high irradiation (Table 2).

### 2.4. Effect of Blue Light Acclimation on the Growth of Vanilla Orchid

#### 2.4.1. BL-Acclimation Enhances Growth and Induces Chloroplast Avoidance

**Figure 3:**
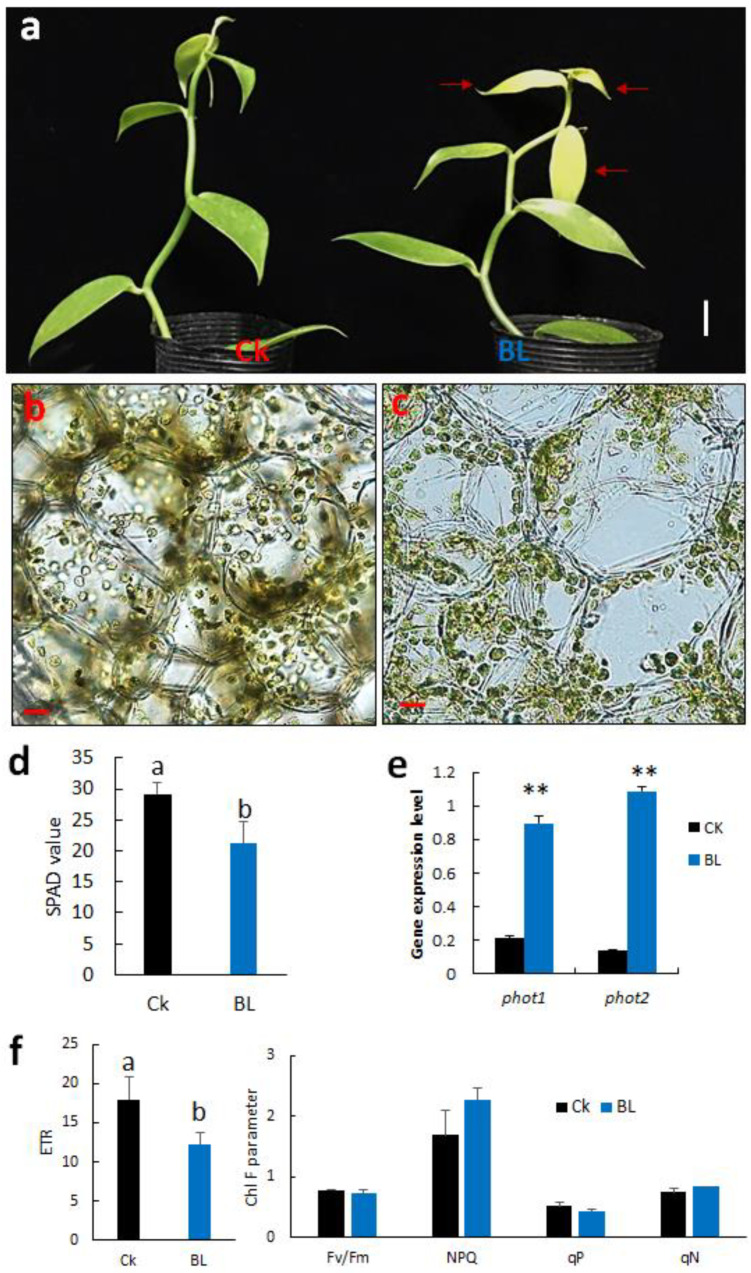
Effect of BL-acclimation on vanilla orchid growth, chloroplast movement, gene expression, and Chl F capacity. (**a**) BL-acclimation for 12 days (BL) affected vanilla orchid growth. Ck, vanilla grown in a greenhouse without BL pretreatment. BL100 treatment for 12 days showed some signs of photobleaching (right panel). Red arrows indicate the L3 has photoinhibition phenotype. Bars, 2 cm. Cross-section of the third newly established leaf (L3) showed that chloroplasts were distributed randomly in Ck (**b**), but chloroplasts moved to the anticlinal (side) walls after BL-acclimation (**c**). Bars, 20 μm (**b**,**c**). (**d**) BL-acclimation reduced SPAD reading due to chloroplast avoidance. (**e**) BL-acclimation upregulated the gene expression levels of blue-light photoreceptors, *phot1* and *phot2*. (**f**) BL-acclimation altered Chl F capacity. ETR, electron transport rate; Fv/Fm, nonphotochemical quenching (NPQ); qP, photochemical quenching of fluorescence; qN, nonphotochemical quenching of fluorescence. Different letters indicate significant differences among treatments at *p* < 0.05, according to Duncan’s multiple range test (DMRT). Bar, SD of three biological replicates.

We performed BL-acclimation of vanilla orchid at 100 μmol m^−2^s^−1^ for 12 days, and some mild photobleaching symptoms were observed in the young leaves, including L3 (Figure 3a, right panel). SPAD data indicated that BL-acclimated vanilla orchid apparently reduced chlorophyll content (Figure 3d) and caused chloroplast avoidance to the side of the cell (Figure 3c). For the control without BL-acclimation, chloroplasts were distributed randomly in the mesophyll cells (Figure 3b). qRT-PCR data indicated that BL-acclimation significantly upregulated transcripts of blue-light photoreceptors, *phot1* and *phot2* (Figure 3e). Results of Chl F indicated that BL-acclimation significantly decreased ETR and slightly increased NPQ but with no statistically significant difference at the 5% level (Figure 3f).

#### 2.4.2. BL-Acclimation Increases Antioxidant Capacity and Sugar Content

**Figure 4:**
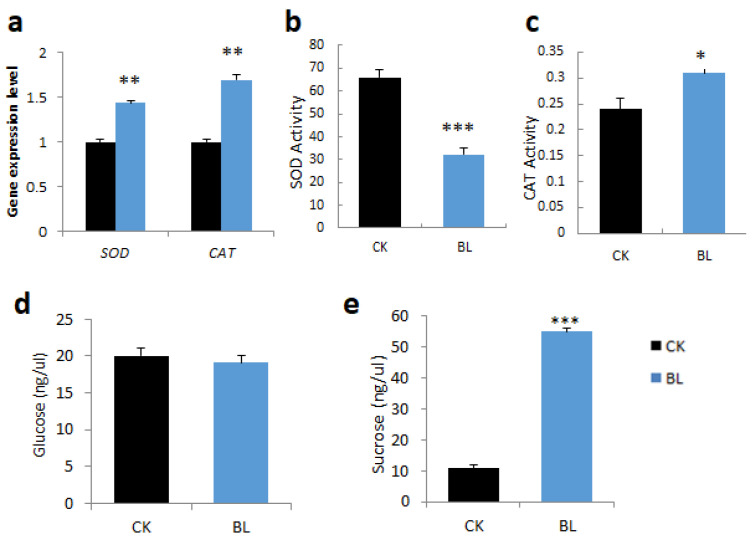
BL-acclimation altered antioxidant transcript expression levels, antioxidant enzyme activities, and sugar content of vanilla orchid. BL-acclimation altered gene expression patterns of *SOD* and *catalase* (**a**), SOD enzyme activity (**b**), catalase enzyme activity (**c**), glucose content (**d**), and sucrose content (**e**). Significant differences in comparison with the control without BL-acclimation (BL) are indicated with an asterisk. Statistical significance was determined by a one-way analysis of variance (ANOVA), followed by Dunnett’s test. * *p* < 0.05, ** *p* < 0.01, *** *p* < 0.001. Error bars represent the standard error of the mean (*n* = 3).

L3 leaf samples of vanilla orchid, with or without BL-acclimation for 12 days, were collected, and then the gene expression pattern, antioxidant enzyme activities, and sugar content were determined. BL-acclimation upregulated SOD and catalase transcript levels (Figure 4a). However, BL-acclimation reduced SOD activity (Figure 4b) but significantly increased catalase activity (Figure 4c). Glucose content was not much changed in L3 after BL-acclimation (Figure 4d), but it contained higher sucrose than the control without BL-acclimation (Figure 4e).

#### 2.4.3. BL-Acclimation Vanilla Orchid Altered Gene Expression Patterns

**Figure 5:**
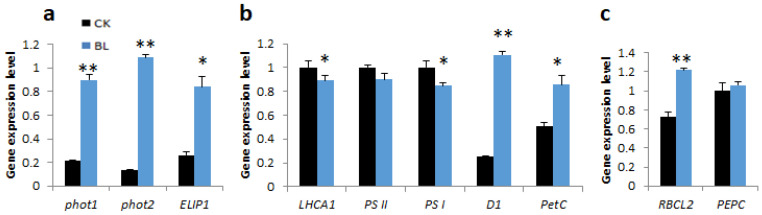
Blue-light-acclimation altered gene expression patterns of vanilla orchid. (**a**) Light responsive genes. (**b**) Marker genes associated with photosystem I and II. (**c**) Genes involved in the Calvin cycle pathway. RNA samples were collected from vanilla orchid L3 after BL-acclimation or not for 12 days. Ck, without BL-acclimation; BL, with BL-acclimation for 12 days. Error bars indicate the SD of the mean from three bioreplicates. Statistical significance was determined by a one-way analysis of variance (ANOVA), followed by Dunnett’s test. * *p* < 0.05, ** *p* < 0.01.

BL-acclimation of vanilla orchid significantly upregulated transcript levels of blue-light-responsive genes such as *phot1*, *phot2*, and the *early light-induced protein 1* (*ELIP1*) (Figure 5a). BL-acclimated plants showed slight downregulation of a light-harvesting complex gene (*LHCA1*), *PSII*, and *PSI*, but significantly upregulated transcript levels of *D1* and *PetC* (Figure 5b). Transcripts of *Rubisco* (*RBCL2*) and *PEPC* were also upregulated after BL-acclimation for 12 days (Figure 5c).

### 2.5. BL-Acclimation Increases Photoprotection of Vanilla Orchid

#### 2.5.1. BL-Acclimation Enhances Growth and Increases Chl F Capacity

**Figure 6:**
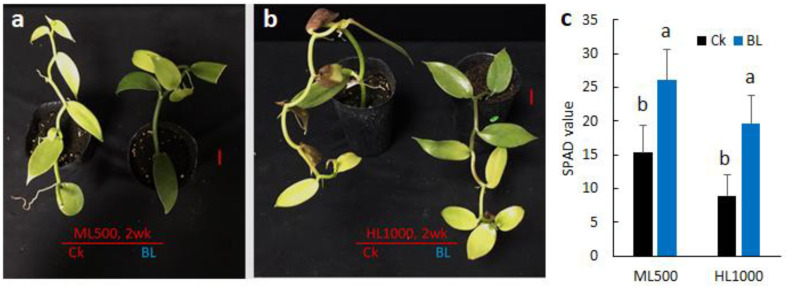
Blue-light-acclimation enhanced vanilla orchid growth after exposure to high irradiation. (**a**) Vanilla exposed to moderately high irradiation of 500 μmole m^−2^s^−1^ (ML500). (**b**) Vanilla exposed to high irradiation of 1000 μmole m^−2^s^−1^ (HL1000). BL, plant after BL-acclimation (BL-Ac) for 12 days. Red scale bars, 2 cm (**a**,**b**). Growth chamber temperature was at a constant 26 °C, 12 h photoperiod. (**c**) SPAD reading of vanilla orchid L3 after exposure to high irradiations of ML and HL for two weeks. Different letters indicate significant differences among treatments at *p* < 0.05, according to Duncan’s multiple range test (DMRT).

Vanilla orchids, with or without BL-acclimation, were exposed to moderately high irradiation (ML500) and high irradiation (HL1000), respectively. After high irradiation for two weeks, vanilla orchid with BL-acclimation had better growth and biomass (Figure 6a,b, and Figure S3) and higher chlorophyll content than the control without BL-acclimation (Figure 6c). Results of the Chl F parameters indicated that Fv/Fm, ETR, NPQ, and qP were significantly different between ML500 and HL1000. Plants with or without BL-acclimation were significantly different for Fv/Fm and qP. Light × BL-acclimation interaction showed a significant difference for Fv/Fm, NPQ, and qP (Table 3). When vanilla orchid was exposed to the moderately high irradiation of ML500, none of the Chl F parameters showed significant differences (*p* < 5%) between those with or without BL-acclimation. However, when exposed to HL1000, the vanilla orchids that had been BL-acclimated sustained significantly higher Chl F capacity for Fv/Fm, ETR, NPQ, qP, and qN than the control without BL-acclimation (Table 3). Clearly, BL-acclimation enhanced vanilla orchid’s acquired photoprotection under high light stress conditions.

#### 2.5.2. Effect of BL-Acclimation on Antioxidative Enzymes and Sugar Content under High Irradiation

**Figure 7:**
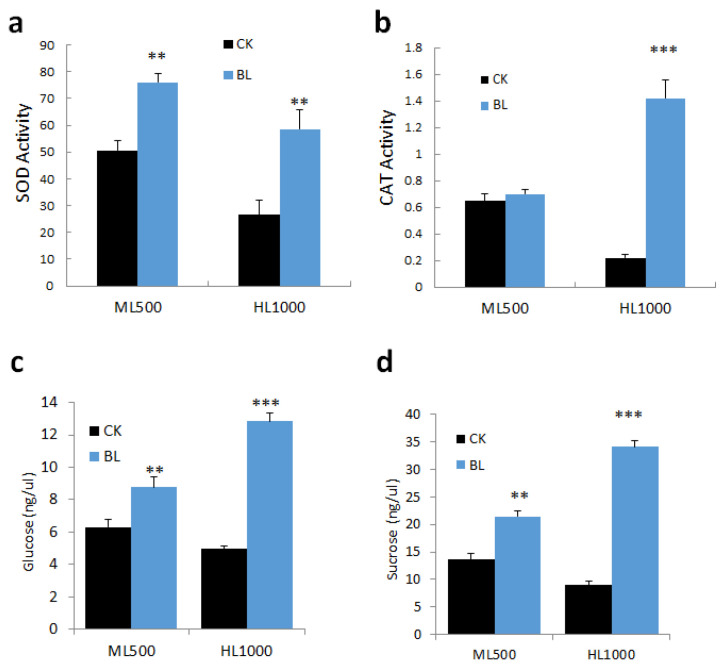
Blue-light-acclimation altered ROS accumulation, antioxidative enzyme activity, and sugar content after exposure to high irradiation. (**a**) Superoxide dismutase (SOD) activity. (**b**) Catalase (CAT) activity. CAT, catalase activity (mU/mL). (**c**) Glucose content. (**d**) Sucrose content. Statistical significance was determined by a one-way analysis of variance (ANOVA), followed by Dunnett’s test. ** *p* < 0.01, *** *p* < 0.001. Error bars represent the standard error of the mean (*n* = 3).

Vanilla orchids were treated with or without BL-acclimation then exposed to ML500 and HL1000 irradiation, respectively. After irradiation for two weeks, L3 leaves were collected and SOD and CAT enzyme activities were detected. Glucose and sucrose content were also compared in the control and BL-acclimated vanilla orchids. Results indicated that BL-acclimation significantly increased antioxidant enzyme activity of SOD after exposure to ML500 or HL1000 (Figure 7a) and CAT activity at HL1000 (Figure 7b). BL-acclimation also significantly increased glucose and sucrose content (Figure 7c,d).

#### 2.5.3. BL-Acclimation Alters Gene Expression Patterns under High Irradiation

**Figure 8:**
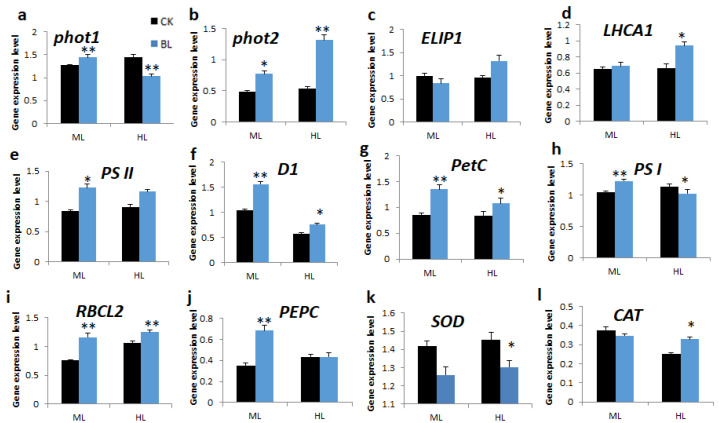
Quantitative RT-PCR showed that BL-acclimation altered gene expression patterns of vanilla orchids. RNA samples were collected after exposure to moderate light at 500 μmol m^2^s^−1^ (ML) or high light at 1000 μmol m^−2^s^−1^ (HL) for two weeks. Error bars indicate the SD of the mean from three bioreplicates. Statistical significance was determined by a one-way analysis of variance (ANOVA), followed by Dunnett’s test. * *p* < 0.05, ** *p* < 0.01.

Leaf samples of vanilla orchids, with and without BL-acclimation for 12 days, were exposed to ML500 and HL1000 for two weeks, respectively, and then L3 leaf samples were collected, RNA was isolated, and qRT-PCR was performed. Results indicated that the BL-acclimation of vanilla only slightly increased *phot1* mRNA at ML500 (Figure 8a) but significantly upregulated *phot2* under ML500 and HL1000 (Figure 8b). *ELIP1* transcript was upregulated after BL-acclimation and then exposed to HL1000 irradiation (Figure 8c). It was found that BL-acclimation upregulated transcripts of marker genes associated with photosystem II such as *LHCA1*, *PS II*, *D1*, and *PetC* (Figure 8d–g). *PS I* transcript was upregulated for the BL-acclimated orchid at ML500 but not under the extremely high light of HL1000 (Figure 8h). Under ML500, transcripts of *Rubisco* (*RBCL2*) and *PEPC* were expressed at higher levels in those vanilla leaves pretreated with BL-acclimation than the control without BL-acclimation (Figure 8i,j). BL-acclimated vanilla upregulated *CAT* mRNA after HL1000 exposure (Figure 8l).

## 3. Discussion

### 3.1. Blue Light Mediates Chloroplast Avoidance in Vanilla Orchid

This study showed that chloroplast movement of vanilla orchid is dependent on blue light (Figure 1a), similar to a report for *Phalaenopsis aprodite* (moth orchid) [12] and other studies [23,24]. A SPAD chloroplast movement assay indicated that young leaves (L3) of vanilla orchid are more sensitive to BL stimuli than mature leaves (LM) because L3 has a larger range of SPAD (Figure 1c,d). This study showed that when vanilla is exposed to BL >20 μmol m^−2^s^−1^, the young and mature leaves both started to exhibit chloroplast avoidance to reduce photoinhibition. Obvious chloroplast avoidance movement of vanilla was found when it was exposed to high BL irradiation of 100 μmol m^−2^s^−1^ (Figure 1c,d). Young vanilla leaves have higher *phot1* and *phot2* expression than the mature leaves (Figure 1e,f), implying that phototropins are preparing to respond to the external light fluctuations. This study also found that *phot1* and *phot2* act together in regulating the chloroplast movement of vanilla, similar to moth orchid [12]. Although in Arabidopsis, the chloroplast avoidance response is exclusively mediated by phot2 [24], Atphot2 is the key player involved in the rapid reorganization of cp-actin filaments that causes chloroplasts to change direction rapidly [25].

### 3.2. Vanilla Orchid is Tolerant to Moderately High Temperature but Sensitive to High Irradiation

The light response curve of Chl F parameters showed that vanilla orchid young leaf has the highest photochemical quenching of fluorescence (qP) under the low light condition (100 μmol m^−2^s^−1^) but started decreasing when light is higher than 150 μmol m^−2^s^−1^. When vanilla was exposed to light higher than 300 μmol m^−2^s^−1^, qP significantly reduced to one-third of that at the irradiation of 100 μmol m^−2^s^−1^. Vanilla significantly lost ETR and qP when exposed to the high light of 1000 μmol m^−2^s^−1^ (Figure 2a,c). However, vanilla ETR showed no significant difference at light intensity <500 μmol m^−2^s^−1^ but almost completely lost ETR at 1000 μmol m^−2^s^−1^ (Figure 2a). Surprisingly, under the moderately high temperature of 33 °C, vanilla has maximum ETR and a high amount of qP but a lower amount of NPQ (Figure 2d–f). These results indicate that under the moderately high temperature of 33 °C, vanilla has high ETR and qP capacities and no need to release excess energy as heat via the NPQ. These findings suggest that vanilla orchid is adapted to the moderately high temperature zone of tropical and subtropical areas through enhanced photosynthesis and thus has better growth. This study shows that similar to moth orchid [22], vanilla orchid is also sensitive to high irradiation. Hence, reduced light intensity is necessary for sustaining a vanilla orchid. Therefore, tree shading or a shaded screen house is a common practice in vanilla production to eliminate photoinhibition damage [4,5]. This study detected the Chl F parameters of vanilla under precisely controlled conditions (light intensity and temperature) and proposes suitable light regimes (100 to 150 μmol m^−2^s^−1^) and temperature (33 °C) that may enhance vanilla photosynthesis efficiency and growth. However, under field plantation, the temperature is usually higher than 36 °C, and high irradiation fluctuates; hence, incorporating high temperature and high irradiation tolerant traits together in a vanilla orchid breeding program may boost crop production.

### 3.3. Impact of Blue Light Acclimation on Vanilla Growth

#### 3.3.1. BL-Acclimation Causes Chloroplast Avoidance to Reduce Photoinhibition

Photoinhibition happens when plants are exposed to excess light above PAR for photosynthesis. It has been reported that BL pretreatment induced photoacclimation and thus assisted plants tolerance to excess light photodamage [26,27,28]. Our previous study indicated that BL100 upregulated *phototropins*, significantly induced chloroplast avoidance movement [12], and induced photoacclimation in *Phalaenopsis aphrodite* (moth orchid) [22]. Moreover, BL-acclimation improves plant growth due to high BL triggering specific biochemical and physiological processes, causing better acclimation and recovery of plants to light stress [22,29]. This study showed that BL100 irradiation of vanilla orchid for 8 or 12 days is sufficient to acquire photoacclimation, as shown in the significant increment of ETR, NPQ, and qP capacities after prolonged blue light treatment (Table 1). We demonstrated that after BL-acclimation for 12 days, vanilla orchid significantly upregulated *phot1* and *phot2* transcripts and mediated chloroplast avoidance movement; thus, chloroplasts moved to the side of cells (Figure 3c), causing a decrease of light-harvest antenna size, and ETR was decreased (Figure 3f). A reduction in the photosynthesis rate, with minimized chloroplast damage, is essential for photosynthesis recovery. It is reported that photosynthesis is more vulnerable to damage in the absence of chloroplast avoidance movement [30].

#### 3.3.2. BL-Acclimation Reduces ROS and Enhances Antioxidants

Anthocyanin pigments reduce the photoinhibition and photobleaching of chlorophyll under high light stress conditions [31]. However, this study showed that vanilla orchid exposure to high irradiation or after BL-acclimation did not accumulate anthocyanins, unlike moth orchid, which accumulates anthocyanin in the young leaves and root tip [22]. Lack of this pigment production may cause vanilla orchid to be more sensitive to photoinhibition when BL100 acclimation is conducted for 12 days, as shown by some bleaching in the young leaves (Figure 3a); after exposure to HL1000, the control plant, without BL-acclimation, showed severe leave wilting symptoms (Figure 6b).

It is well known that ROS are important regulatory molecules for plant growth and development, which determine plant acclimation ability under stress. In addition, ROS is produced during photosynthesis electron transport. If ROS molecules exceed the antioxidant system’s capability to detoxify them, the ROS imbalance will reduce the photosynthesis rate [8]. Moreover, high blue light induced H_2_O_2_ in *Arabidopsis* plasma membrane and chloroplasts and regulated chloroplast avoidance [32]. This study showed that BL-acclimation enhances vanilla tolerance to high irradiation. BL-pretreatment generates a “mild stress”, and the vanilla orchid acquires some level of stress tolerance toward subsequent high irradiation challenge. Vanilla produced more ROS molecules but upregulated *SOD* and *CAT* mRNAs and turned on antioxidative enzyme activities to carry out the ROS scavenging process to detoxify ROS (Figure 4). This study and a previous study [22] found that sometimes, SOD enzyme activity is not correlated with *SOD* gene expression in orchids (Figure 4d). It has been reported that ROS scavenging genes are not relevant to antioxidative enzyme activities, and this may be due to the increase in transcription level not being translated into proteins in plants under conditions of stress [33]. Besides ROS scavenging, plants also turn on other photoprotective mechanisms, such as inducing enzymes involved in antioxidants, changes in light-harvesting capacity, and energy-dissipating mechanisms in PSII or PSI [7]. Blue light triggers specific biochemical and Chl F parameters, resulting in better recovery when cucumber responds to high UV light stress [34].

#### 3.3.3. BL-Acclimation Increases Photosynthesis-Related Genes under High Light Stress

This study showed that after exposure to high irradiation stress conditions, BL-acclimatized vanilla maintained higher ETR and NPQ capacities than the control without BL-acclimation (Table 3). Moreover, BL-acclimated vanilla orchid showed increased SOD and CAT enzyme activities to reduce ROS (Figure 7a,b). Of note, under conditions of high irradiation, vanilla with BL-pretreatment showed upregulated *PSII*, *D1*, *PetC*, *Rubisco*, and *PEPC* transcripts (Figure 8). Rubisco activity is regulated by light [35]. Glucose and sucrose contents are increased (Figure 7c,d), and, subsequently, BL-acclimated vanilla orchids had higher biomass (Figure 6 and Figure S3). It has been reported that transgenic plants overexpressing *D1 protein* had increased photosynthesis efficiency and enhanced crop yield [16]. In addition, overexpressing RiskeFes protein (*PetC*), a component of Cyt b_6_f complex, induces D1 and increases the photosynthesis rate and plant biomass [17]. We found that transcripts of *D1* and *PetC* are upregulated during BL-acclimation as well as after high irradiation exposure (Figure 8f,g). Manipulation of light-harvesting capacity by the PSII reaction center and photochemical quenching are effective strategies to increase crop photosynthetic efficiency [36]. An increase in blue light percentage is associated with an increase in leaf biomass, chlorophyll content, photosynthesis rate, and stomatal conductance of cucumber [37] and lettuce [38]. Overall, the combination of several signals and coordination with other environmental stress signal transduction pathways is important for plant acclimation and tolerance to excess light [9].

## 4. Materials and Methods

### 4.1. Plant Materials and Experimental Design

Potted vanilla orchid plants (*Vanilla planifolia*) with five to six leaves were purchased from Vanilla Knight Co. (Puli, Taiwan) and planted in the AS-BCST greenhouse with 50% shading, at a temperature of 26 ± 3 °C. In order to know the light and temperature requirement for vanilla orchid, we carried out a light response experiment (100, 150, 200, 300, 400, 500, and 1000 μmol m^−2^s^−1^) and studied the effect of temperatures (21, 24, 26, 30, 33, and 36 °C) on Chl F parameters of vanilla. Each treatment was conducted on three plants.

To identify how many days is sufficient to acquire BL-acclimation in vanilla orchid, we treated vanilla plants with BL100 in a constant 26 °C growth chamber for 4, 8, and 12 days, and the control was without BL-acclimation. Each treatment was conducted on three plants. To further study the effect of BL-acclimation protection on vanilla orchid exposure to high irradiation, vanilla plants were separated into two parts (control and BL-acclimation). A total of 18 plants were BL-acclimatized in a growth chamber set at a constant 26 °C and continuous irradiation with light-emitting diode (LED) blue light (BL, λmax = 450 nm) at 100 μmol m^−2^s^−1^. BL-acclimation was set without a dark period in order to avoid other acclimation factors such as dark or low temperature. After BL-acclimation for 12 days, three plants were used to perform chlorophyll fluorescence measurement, and three plants were collected to take samples for RNA, ROS, and biochemistry analysis. The other six plants were exposed to moderate irradiation for two weeks in a growth chamber (F-1300, Taiwan Hipoint Co., Kaohsiung, Taiwan) under LED white light at 500 μmol m^−2^s^−1^ (ML500, containing 80 μmol m^−2^s^−1^ BL) or high irradiation of 1000 μmol m^−2^s^−1^ (HL1000, containing 160 μmol m^−2^s^−1^ BL). After high irradiation for two weeks, chlorophyll fluorescence was measured in three plants, and three plants were collected for RNA, ROS, and biochemical analysis. Growth chambers were set at a constant 26 °C, 12 h light/12 h dark period, whilst the other set of vanilla plants were kept in white light without BL-acclimation and then exposed together with the BL-acclimated plants to ML500 and HL1000, respectively.

### 4.2. Chloroplast Movement Using SPAD Measurement

To assay nondestructive chlorophyll content, we used a soil plant analysis development (SPAD) chlorophyll meter (model SPAD-502). To detect the chloroplast movement of vanilla, vanilla plants were dark-adapted overnight, and the new fully expanded leaf (the 3rd leaf, L3) and mature leaf (LM) were used to measure SPAD, following the procedures (Figure S2) and protocol described previously [12]. Briefly, vanilla orchid plants were dark-adapted for 16 h, then exposed to different blue light intensities of 5, 10, 20, 50, and 100 μmol m^−2^s^−1^, respectively, and SPAD data of the L3 leave were measured every hour for a total of five hours. SPAD data were normalized to the initial data after dark-adaptation.

### 4.3. Measurement of Chlorophyll Fluorescence Parameters

Chlorophyll fluorescence (Chl F) parameters were measured using a LiCOR 6800 system (Li-Cor, Inc., Lincoln, NE, USA) equipped with a fluorescence leaf chamber 6800-01A (Li-Cor, Inc., Lincoln, NE, USA) and 2 cm^2^ leaf area aperture for the Chl F measurements. Leaf chamber temperature was set at 26 °C, and CO_2_ concentration was set at 400 ppm. L3 leaves were covered with aluminum foil and dark-adapted more than 2 h before measuring the parameters of chlorophyll fluorescence. A saturating actinic light pulse of 8000 μmol m^−2^s^−1^ for one second was applied to measure the maximal fluorescence. Actinic light is composed of 90% red light (625 nm) and 10% blue light (470 nm). Data of Fv/Fm, electron transport rate (ETR), nonphotochemical quenching (NPQ), photochemical quenching of fluorescence (qP), and nonphotochemical quenching of fluorescence (qN) were recorded and analyzed. Each treatment has three bioreplicate measurements.

### 4.4. Measurement of Antioxidant Enzyme Activities and Sugar Content

Vanilla orchid newly expanded L3 leaf samples, with or without BL-acclimation for 12 days, were exposed to ML500 or HL1000 for two weeks and then collected and stored at −80 °C freezer. A total of 100 mg of frozen leaf samples were homogenized. SOD enzyme activity was determined using a Superoxide Dismutase (SOD) Activity Colorimetric Assay Kit (BioVision #K335-100), following the instruction of the manufacturer. SOD enzyme activity was calculated based on the percentage inhibition after 20 min of incubation at 37 °C. For detection of catalase (CAT) enzyme activity, 100 mg frozen leaf samples were homogenized and determined using a Catalase Activity Colorimetric/ Fluorometric Assay Kit (BioVision, #K773-100), as described previously [22].

Vanilla orchid L3 leaves, with or without BL-acclimation for 12 days, which had then been exposed to moderate light (ML500) or high light (HL1000) irradiation for two weeks, were collected. A total of 100 mg of leaf samples were homogenized, 500 μL of 1 × PBS buffer was added, and then the mixture was incubated for 30 min at 37 °C. Then, glucose and sucrose concentrations were determined using a Glucose and Sucrose Colorimetric/Fluorometric Assay Kit (Sigma #MAK013), following the instructions provided by the manufacturer.

### 4.5. Gene Expression Analysis

L3 leaf samples from vanilla orchids, with or without BL-acclimation for 12 days, which had then been exposed to ML500 or HL1000 for two weeks, were collected. Total RNA was isolated and DNase treated, then 1 μg RNA was reverse-transcribed to 1st cDNA using the M-MLV reverse transcriptase cDNA synthesis kit (Promega Co., Madison, WI), and then qRT-PCR was performed for 40 cycles with KAPA SYBR FAST qPCR Master Mix (2×; Kapa Biosystems, Woburn, MA, USA), as described previously [12]. Primers used in this study for qRT-PCR are recorded in Table S1. The qRT-PCR reaction was performed in triplicate on a C1000TM Thermal Cycler (Bio-Rad). Relative gene expression level was normalized to a housekeeping gene, *Ubiquitin* (VPTC006466), as a control.

### 4.6. Statistical Analyses

Statistical analyses were performed using Statistical Analysis System (SAS) software version 9.4, using the analysis of variance (ANOVA) procedure, followed by Duncan’s multiple range test. *p*-values of less than 0.05 were considered statistically significant. Dunnett’s test was used to compare the difference between BL-acclimation and the control without BL-acclimation.

## 5. Conclusion and Perspective

Collectively, this study demonstrates that pretreated vanilla under strong blue light at 100 μmol m^−2^s^−1^ for 12 days induces photoacclimation in this orchid species. Blue light triggers biochemical and physiological processes that confer better recovery when vanilla orchids are exposed to high irradiation. Vanilla blue-light photoreceptors, phototropins, sense the high fluence rate of blue light and upregulate *phot1* and *phot2* gene expression and mediate chloroplast avoidance in order to reduce photodamage. BL-acclimation enhances antioxidant activities and decreases ROS toxicity in vanilla orchid, resulting in increases in the transcript levels of *PSII*, *D1*, and *PetC*, enhancing PSII repair and sustaining the photosynthesis rate under conditions of high irradiation. Hence, acclimating vanilla orchids with BL before transplantation to the field might enhance photoprotection and improve its growth and production. In the future, the knowledge gained from this study may help to manipulate the photosynthesis efficiency of vanilla orchid and improve its resilience to environmental stress. In addition, BL-acclimation may be applied to other crops to hardening seedlings, increase field survival, and increase crop yields.

## Figures and Tables

**Table 1:** ANOVA shows the mean square of relative Soil Plant Analysis Development (SPAD) data of young (L3) and mature leaves (LM) of vanilla orchid exposure to various blue light intensity and different time courses.

Source	df	L3	LM
Model	29	0.0665 ***	0.0204 ***
BL	5	0.3737 ***	0.1057 ***
Time	4	0.0012 ns	0.0062 ns
BL*Time	20	0.0027 ns	0.0019 ns
Error	120	0.0035	0.0027
Total	149		

*** *p* < 0.001; n.s., no significant difference at *p* < 0.05.

**Table 2:** Effect of various blue light (BL)-acclimation time courses on Chl F parameters of vanilla orchid.

BL Day	Light		Fv/Fm		ETR		NPQ		qP		qN	
0 day	150		0.74	a	9.50	a	2.05	b	0.32	a	0.81	b
	500		0.75	a	7.08	ab	2.59	ab	0.08	b	0.85	ab
	1000		0.74	a	5.35	b	2.82	a	0.03	b	0.88	a
4 day	150		0.74	a	15.75	a	2.50	a	0.60	a	0.86	b
	500		0.73	a	16.97	a	3.06	a	0.22	b	0.90	a
	1000		0.72	a	7.82	b	3.03	a	0.06	c	0.90	a
8 day	150		0.70	a	19.62	a	2.13	b	0.65	a	0.81	b
	500		0.70	a	16.63	a	3.11	a	0.24	b	0.90	a
	1000		0.72	a	16.25	a	3.36	a	0.13	c	0.91	a
12 day	150		0.71	a	17.02	a	2.53	b	0.64	a	0.85	b
	500		0.71	a	16.08	a	3.14	ab	0.23	b	0.90	a
	1000		0.68	a	10.32	b	3.30	a	0.08	c	0.92	a
		df										
BLday		3	**		***		*		***		***	
Light		2	n.s.		***		***		***		***	
BLday × Light		6	n.s.		*		n.s.		***		n.s.	

Statistical significance was determined by the analysis of variance (ANOVA) procedure, followed by Duncan’s multiple range test. Means in the row followed by the same letter indicates no significant difference between treatments (*p* < 0.05). Each treatment has three bioreplicates. * *p* < 0.05, ** *p* < 0.01, *** *p* < 0.001; n.s., no significant difference at *p* < 0.05.

**Table 3:** Comparison of vanilla orchids with or without BL-acclimation on Chl F parameters after exposure to ML500 and HL1000 conditions for two weeks.

Light	BL_Ac	df	F/vFm		ETR		NPQ		qP		qN	
ML500	BL		0.59	a	7.03	a	2.03	a	0.36	a	0.87	a
	CK		0.61	a	7.99	a	2.12	a	0.34	a	0.85	a
HL1000	BL		0.42	a	5.65	a	1.98	a	0.35	a	0.89	a
	CK		0.08	b	−0.56	b	0.58	b	−0.34	a	0.81	a
Light		1	***		*		*		*		n.s.	
BL_Ac		1	*		n.s.		n.s.		*		n.s.	
Light × BL_Ac		1	*		n.s.		*		*		n.s.	

Ck, vanilla orchid without BL-acclimation. Different letters indicate significant differences among treatments at *p* < 0.05, according to Duncan’s multiple range test (DMRT). Means in a row followed by the same letter indicate no significant difference between treatments (*p* < 0.05). Each treatment had three bioreplicates. * *p* < 0.05, ***, *p* < 0.001; n.s., no significant difference at *p* < 0.05.

## Supplementary Materials

Supplementary materials can be found at https://www.mdpi.com/1422-0067/21/21/8022/s1. Table S1: Primers used in this study. Figure S1: High light irradiation in the screen house caused sunburn symptoms on the leaves of vanilla orchids. Figure S2: Procedures to measure chloroplast movement using a SPAD meter. Figure S3: Vanilla orchids exposed to high irradiations of ML500 or HL1000 for two weeks showed severe photoinhibition but BL-acclimation reduced the photodamage. (a) ML500 (b) HL1000. Bars, 2 cm.

## Abbreviations

BL	Blue light
ML	Moderate light
HL	High light
PSI	Photosystem I
PSII	Photosystem II
Fv/Fm	Maximal quantum efficiency of PSⅡ photochemistry
ETR	Electron transport rate
NPQ	Nonphotochemical quenching
qP	Photochemical quenching of fluorescence
qN	Nonphotochemical quenching coefficient
SOD	Superoxide dismutase
CAT	Catalase
ROS	Reactive oxygen species
