# Supplementary material for: Blue Light Mediates Chloroplast Avoidance and Enhances Photoprotection of Vanilla Orchid
Source: Int J Mol Sci. 2020 Oct 28;21(21):8022. doi: 10.3390/ijms21218022 (PMC7663427; doi:10.3390/ijms21218022)
Supplement: Supplementary file 1 [file ijms-21-08022-s001.pdf]

Supplementary materials

Table S1. Primers used in this study.

Name	Oligo 5′→ → 3′	Contig
Ubi-F	TGAACTCCATCGCCTTCCTCTTC	VPTC006466
Ubi-R	TGAAGCATGGCATCAATTTC	
VnPHOT1-F	ATGGAAGAGAAGGAGGAACGGC	VPTC017750
VnPHOT1-R	TGTCGGACAGCACCTGGATC	
VnPHOT2-F	CAATAGAACTCGACCATAAATCTTAC	VPTC16000
VnPHOT2-R	CTTGCATGTCCAATCCTGGT	
Vn_ELIP1-F	CGTTCGACATCTTGCCTTGCC	VPTC001069
Vn_ELIP1-R	CAGAGATTGACAGATGAGACCACATG	
Vn_LHCA1-F	CAAGGTGAAGGAGATTAAGAACGG	VPTC009056
Vn_LHCA1-R	ATGGATCTGGGAATGATGACGT	
Vn_PSII-F	GCATCAGGAAGGAAGGCTAAGG	VPTC001554
Vn_PSII-R	GCACTGGTATTGTAAACTAGAAGAGCAC	
Vn_D1-F	TGCGACCTTGGATTGCTGTTG	VPTC017296
Vn_D1-R	CCGAAGACACCAGCTACGCC	
VnPetC-F	CCAAGGCACCGTTGTAAGAGG	VPTC016179
VnPetC-R	CGTTTGTTCTTCTCCTTGCCC	
Vn_PSI-F	CGTGCTGAGGATGCCGCTT	VPTC006498
Vn_PSI-R	GAACCGAACTACCACTGGATAACG	
Vn_RBCL2-F	CAATCCCAAGCATGGTTACAATG	VPTC016890
Vn_RBCL2-R	GCCGTATCCTGAGTTGTCCATTG	
Vn_PEPC-F	CAGGTTACCATGTGACGGCGA	VPTC000540
Vn_PEPC-R	CACCTCCTGAGCACTGGAAGC	
Vn_SOD-F	CTCCCGAAGATGAGAATCGCC	VPTC003604
Vn_SOD-R	ATCAGCATGGACAACAACAGCC	
Vn_CAT-F	CGACAACTTCTTCAACGAGAACGA	VPTC009091
Vn_CAT-R	GTCGTAGTGGTTGTTGCGGAAG	

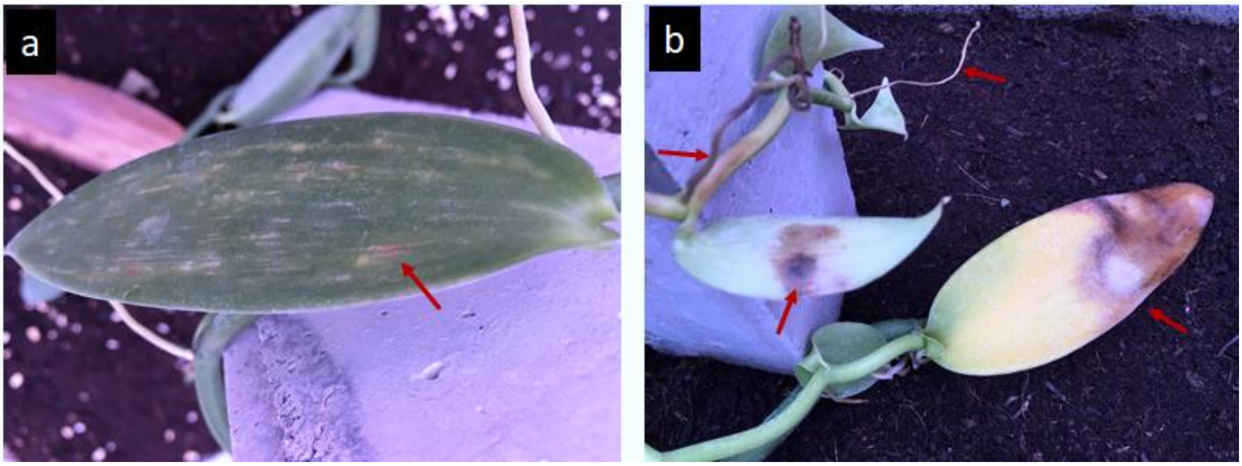

Figure S1. High light irradiation in the screen house caused sunburn symptom on the leaves of vanilla orchids.

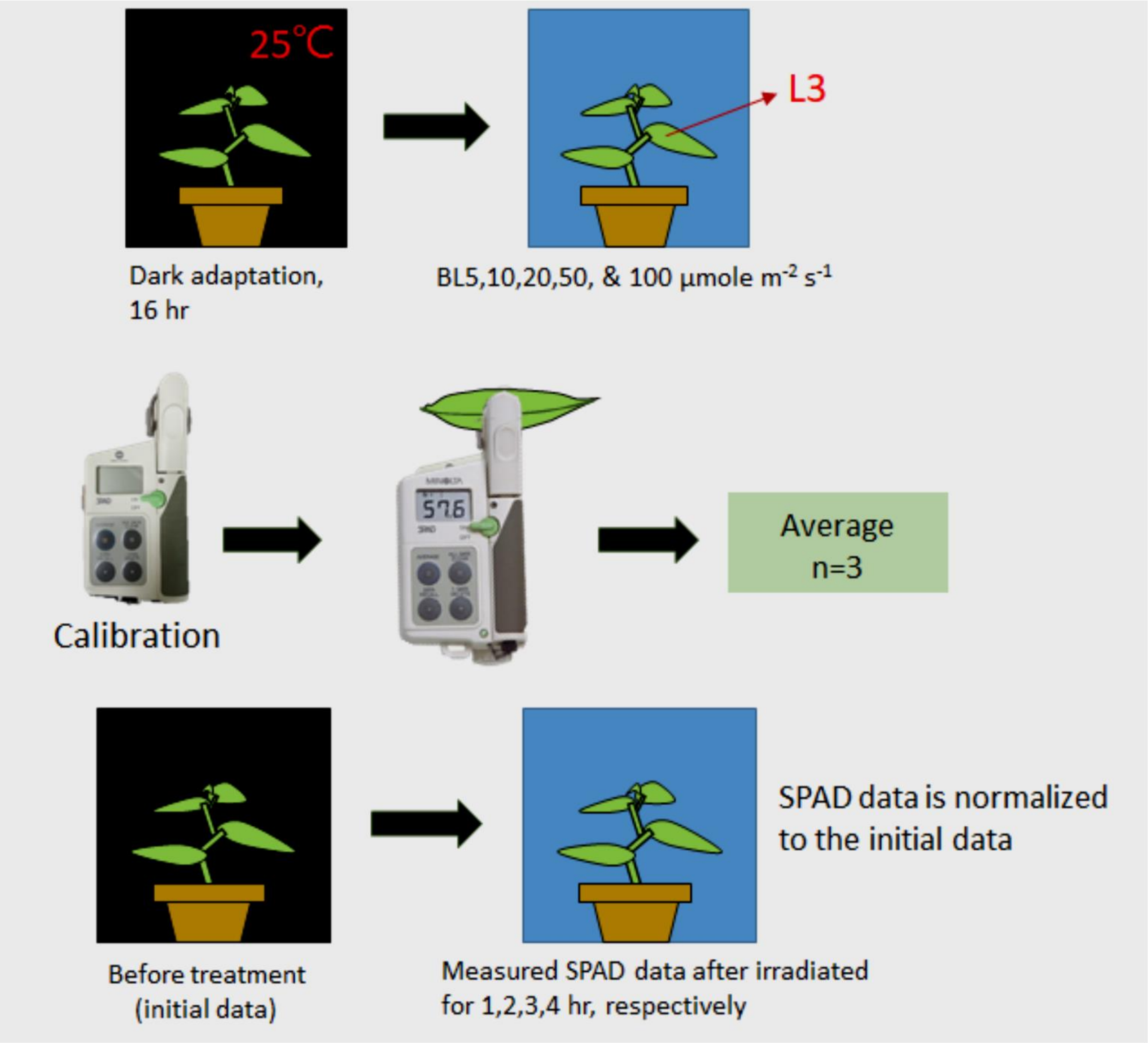

Figure S2. Procedures to measure chloroplast movement using a SPAD meter.

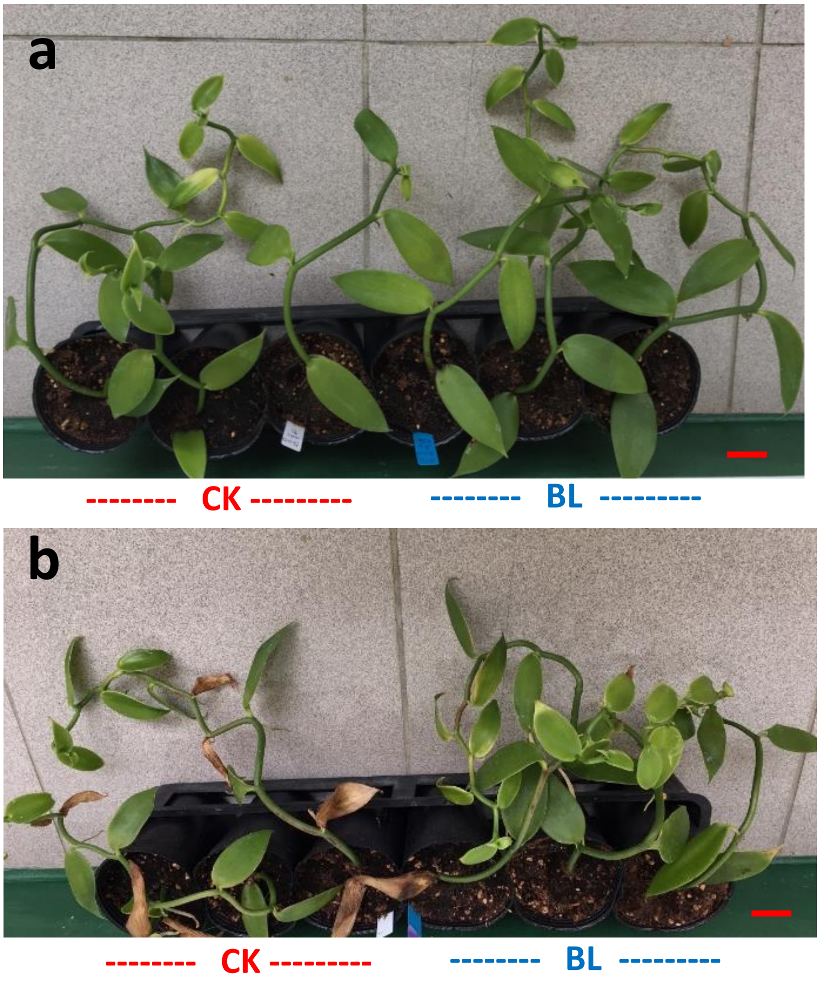

Figure S3. Vanilla orchids exposed to high irradiations of ML500 or HL1000 for two weeks caused severe photoinhibition but BL-acclimation reduced the photodamage. (a) ML500 (b) HL1000. Bars, 2cm.
